# The epistemic advantages of representative deliberation

**DOI:** 10.1371/journal.pone.0353387

**Published:** 2026-07-17

**Authors:** William J. Berger, Daniel J. Singer, Patrick Grim, Aaron Bramson, Bennett Holman, Jiin Jung

**Affiliations:** 1 Department of Philosophy, University at Buffalo, Buffalo, New York, United States of America; 2 Department of Philosophy, University of Pennsylvania, Philadelphia, Pennsylvania, United States of America; 3 Center for the Study of Complex Systems, University of Michigan, Ann Arbor, Michigan, United States of America; 4 Department of Philosophy, Stony Brook University, Stony Brook, New York, United States of America; 5 AI Strategy Center, GA Technologies, Tokyo, Japan; 6 Underwood International College, Yonsei University, Seoul, South Korea; 7 Department of Psychology, Lehigh University, Bethlehem, Pennsylvania, United States of America; Universidade Aberta Departamento de Ciencias Sociais e de Gestao, PORTUGAL

## Abstract

It is widely thought that deliberative quality improves with the number of participants: the more voices in the room, the better the collective judgment. This “wisdom of the crowds” intuition suggests that representative deliberation — in which a subset of deliberators acts on behalf of the larger group — should be epistemically inferior to full, plenary deliberation. We test this using a computational agent-based model in which deliberators exchange evidence for and against a proposition and are evaluated on how accurately their collective beliefs track an objective truth. Varying four conditions — the length of deliberation, the distribution of available evidence, problem difficulty, and agents’ memory capacity — we find that representative deliberation frequently matches or outperforms full deliberation. This advantage does not stem from any superior epistemic ability of the representatives themselves. Rather, it emerges from two structural features of the two-tier process: the selective triage of the strongest available evidence, and the periodic resetting of polarized or entrenched beliefs that a second phase of deliberation enables. Our findings suggest that representative structure can be an epistemic asset rather than a liability — not despite, but because of the constraints it imposes.

## 1. Introduction

It is widely thought that deliberative quality improves with the number of participants—the more voices in the room, the better the collective judgment [1–4]. This “wisdom of the crowds” intuition suggests that representative deliberation, in which only a subset of deliberators acts on behalf of the larger group, should be epistemically inferior to full plenary deliberation. [4–7]. If this view is correct, the collective judgment of deliberative events using full, plenary deliberations should achieve better epistemic results than when representative deliberations pass on recommendations from more expansive lower deliberative bodies to more restrictive higher ones. However, a growing literature on democratic deliberation argues that structured, representative forms of participation can outperform plenary deliberation on key deliberative and democratic dimensions [8,9]. Our research question asks how this plays out. Focusing on the deliberative exchange of evidence, might representative deliberation yield better results than full deliberation? The project here examines whether a hierarchical, representative deliberative process can, under ideal circumstances, lead to better collective judgment than a single, full deliberative body in which all participants deliberate together. We find that the outcomes of representative deliberation can match or even exceed those of full deliberative systems under certain conditions, such as when deliberators face time constraints, possess particularly strong evidence, confront difficult problems, or contend with polarization. Importantly, this is not because representatives themselves are epistemically gifted in our model, but because the structural features of representation itself (1) promote the prioritization of important information and (2) periodically break up entrenched beliefs, providing deliberation an opportunity to reset.

Comparing the epistemic success of full and representative deliberation using observational techniques presents substantial logistical and moral challenges. To circumvent these, we use a computational agent-based model (ABM) to ‘open the hood’ of these two deliberative modes [10–12]. Our approach brings together research in political epistemology [5,7,13,14], theories of political representation [15–20], and deliberative democracy [21–26]. Although some recent work emphasizes the risks and failures of political deliberation [27–32], the successes of deliberative interventions prompt us to probe their mechanics more deeply [33–35].

The paper proceeds as follows: We begin in the next section by outlining the high-level contours and motivation for our models of full and representative deliberation. We detail the deliberation procedures and characterize four key conditions that shape group performance: (a) length of deliberation, (b) evidentiary heterogeneity, (c) problem difficulty, and (d) memory constraints. We then present simulation results, comparing the performance of full and representative deliberation across these four features. Finally, we discuss the structural implications of our findings. We argue that the advantages of representative deliberation here result from the structure of representation itself, rather than any special features or abilities that the representatives themselves might have.

## 2. Motivation and methods

Deliberative events such as minipublics, planning cells, and citizen assemblies can employ different forms of full or representative deliberation. In these institutions, non-elites are brought to deliberate on timely policy issues and offer recommendations to policymakers. They are widely used to better incorporate more diverse perspectives into political decision making and combat declining trust in political institutions [26,34,36–49]. A particularly concrete example of our model of representation can be found in planning cells [36]. Here a number of citizens are chosen at random to offer recommendations on a specific civic proposal and initially educated on the matter by experts. Participants are then broken up into smaller groups and subsequently deliberate independently (without experts) with each group deliberating in parallel. Every group then passes along formal recommendations to “neutral organizers” who then each deliberate further before passing on public recommendations [37]. For instance, in 1987 Germany implemented planning cells around their transition to the ISDN (Integrated Services Digital Network) telecommunications standard. These planning cells deliberated regarding specific policy questions, such as how to implement billing protocols under the new standard [37].

Our paper looks at the mechanisms by which hierarchical, representative deliberative systems, like planning cells, can yield collective judgements that are better than plenary deliberations. Planning cells specifically mirror our model of representation, as in both, members are provided evidence regarding a particular proposition, divided into parallel groups which deliberate independently and then offer summary reasons to a subsequent tier of fewer deliberators.

### 2.1. Model overview

To investigate this question, we use an agent-based model (ABM), following a methodology with a rich history in social science (e.g., [50–52]). An ABM is a computational tool in which individual “agents” — each following a defined set of behavioral rules — interact with one another, generating collective outcomes that can be studied systematically across simulated trials. This approach has proven productive in social research by enabling the study of how group structure shapes collective outcomes. Because real deliberative bodies cannot be easily manipulated experimentally, observational comparisons across formats are often confounded by contextual factors that are difficult to isolate. By running thousands of simulated trials while varying specific parameters — in our case deliberation length, evidentiary heterogeneity, problem difficulty, and memory constraints — in a controlled setting, ABMs allow us to trace the effects of deliberative structure on collective performance in ways that neither experimental nor observational methods can achieve.

In our model, the agents are idealized deliberators. They each begin with a randomly assigned subset of the available evidence (their “reasons”) and follow simple rules for sharing and incorporating reasons they receive. Agents are not programmed with strategic interests, emotional dispositions, differential social status, or other features characteristic of real deliberators; these elements are deliberately abstracted away. This idealization is methodologically intentional: it allows us to evaluate what representative and full deliberation can achieve on structural grounds alone, establishing a theoretical baseline against which more complex models — and real deliberative institutions — can be assessed.

We determine success in the model by measuring how well agents get at a subject-independent truth: the outcome supported by the balance of all of the evidence [53]. Because no agent begins with complete access to that evidence, deliberation is the mechanism by which collective epistemic standing improves. Our central question is whether this convergence is better achieved through full deliberation (“Full”) — in which all participants share evidence together throughout a fixed deliberative period — or through representative deliberation (“Rep”), in which evidence is first pooled within smaller cells before a subsequent tier of representatives continues the process.

Many prominent treatments of epistemic democracy contend that there are objectively right answers to deliberative matters [4,14,54]. While there are many matters that are fiercely divided and where debate only partially serves an epistemic role, even these problems are frequently contingent on questions that have more straightforwardly right or wrong answers [55]. Yet fundamentally, any deliberation requires at least some underlying interpretive agreement to be effective, the elements of which serve as subject-independent truths [35,56–58]. In the most restricted sense of the model here, we can think of it as agents deliberating within a commonly held framework where agents all agree on the direction the evidence points as well as its strength.

Our evidence (or “reasons”) is modeled by numbers. Positive numbers can be thought of pointing in favor of the proposition and negative numbers against. The larger the magnitude of the number the more strongly it supports the contention, for or against. Agents in the model begin with a randomly assigned subset of reasons from a pool of all the available evidence, the balance of which represents the truth. In full deliberation all agents collectively exchange reasons for a fixed length of deliberation. In representative deliberation agents are initially assigned to subgroups (“cells”) before deliberation begins and then deliberate for a fraction of the full fixed period, after which representative agents are created pulling from the strongest reasons of their group. The representative agents of each group then continue to deliberate among themselves until the length of deliberation equals that of the full deliberative mode.

We measure the relative success of full and representative deliberation by measuring the percentage of agents in each mode whose evidence supports the truth at the end of deliberation. In this study, we focus on several important factors that affect how likely a deliberating group is to reach the right answer. Those factors are: (1) how much time is available for deliberation, (2) how widely the evidence is distributed, (3) how difficult the problem is, and (4) how forgetful agents are.

(1) Length of deliberation: Each run of the model has a fixed duration. Each “tick” or step of the model represents one round of deliberation in which one agent shares evidence with others. The more rounds, the longer the deliberative session. In real life some deliberative forums go on for as long as it takes to come to agreement, while in other forums decisions are made after a fixed amount of time has elapsed.
(2) Evidentiary heterogeneity: As we’ll describe in detail below, the quality of reasons available for agents to exchange can be more or less homogeneous. In some cases, which way the evidence points will be determined by a small number of particularly strong relevant pieces of evidence, while in other cases, a large number of weaker reasons will do that work.
(3) Problem difficulty: A proposed advantage of representation is that representative agents tend to be more competent, better able to solve problems than ordinary voters [7]. In our model, every run has different randomly-generated reasons, and the sum of all the reasons—in favor of the proposition and against—can be closer or further from zero. When the balance of the evidence is close to zero, the problem will be more ‘difficult’ since just a few lightweight reasons can have an outsized effect on an agent’s accuracy. We use the model to assess the effect of problem difficulty on the relative performance of the different modes of deliberation.
(4) Memory constraints: Finally, we’ll use the model to assess how different kinds of limitations on how much evidence an agent can retain at once affect the relative performance of the different deliberation modes. We model cases where agents have unlimited memory as well as cases where they can only retain a fixed number of reasons at once.

While there are correspondences between real-world deliberative events and our modeling procedure, our aim here is not to capture the full complexity of actual deliberative processes. Rather we want to understand the high-level dynamics of how deliberative structure shapes collective epistemic success across a range of idealized conditions. Each condition is therefore given a conceptually abstract, operationally precise definition suited to the ABM methodology. So, “deliberation length,” for example, should not be seen as a measure of clock time but simply a comparative measure of the time allocated to deliberation—100 deliberative steps offers much more time than 5 steps. In the same vein, the model represents deliberation as the exchange of evidence, abstracting away other persuasive elements of discussion. Reasons are treated as additive, cleanly able to square with one another. Agents don’t corrupt or alter evidence — they can only forget — and there are no transmission errors. Nor are there principal-agent problems: representative agents don’t impose idiosyncratic prerogatives on the resulting deliberation. Of course, in real-world deliberation, emotional persuasion, status, context, and norms all play a role in how discussions resolve; our model brackets these in order to isolate the structural effects of deliberative organization on epistemic outcomes. While these modeling assumptions idealize certain aspects of real-world deliberation, they also reflect many of the normative justifications for structured deliberative interventions [21,22,34,35,41].

### 2.2. Model procedure

While we laid out the model in general terms above, here we do so more formally. Initially a pool of *r* distinct reasons is generated for and against some proposition. Each reason has a weight and a valence. Weights (positive real numbers) are sampled from a positive distribution with a fixed mean, which can be thought of as the reason’s strength. Reason-weights are initially pulled from a gamma distribution allowing us to hold the expected value of the sampled values constant while adjusting the variance across different runs, corresponding to different levels of evidentiary heterogeneity. We implement this by constructing two parameters, α and β, which determine the mean of the gamma distribution (α/β) and its variance ($\alpha /\beta^{\text{2}}$). Varying α and β allows us to sweep over a range of variances while holding the mean fixed at 1.

Valences are then assigned by randomly multiplying each reason-weight by 1 or −1, thereby creating reasons that can span the real number line. We think of positive values as being in support of a proposition and negative values being against it. The ‘truth’ or ‘correct view’ in a model run is the valence (either positive or negative) of the sum of *all* the *r* generated reasons. So, if *r* = 5 with reasons {−2, 0.23, 0.8, −8, 5.2}, we consider the truth to be negative because the reasons sum to −3.77. While the mean and distributional form are fixed across all our runs, reason-weights are generated at variances ranging from 0.2 to 100 across different model runs. This allows us to measure the effect of evidentiary heterogeneity on the two deliberative modes.

We evaluate the epistemic success of the groups by measuring the percentage of agents on the side of the truth at different time-steps. The idea is that agents are searching for an independent truth (e.g., whether to implement a particular billing practice of the ISDN) by sampling from a distribution of evidence that no individual (or even the whole group) necessarily has complete access to. That being said, as the size of available evidence grows, it becomes highly likely that the group’s belief tracks the truth as the evidence is shared. Our conception of the truth is agent-independent since we treat the truth as the balance of all the generated reasons, whether or not the agents are in possession of that evidence.

In every model run *n* agents are generated and then each randomly assigned *m* reasons (*m* ⊆ *r*) to their “headspace” (body of evidence). Each agent is then duplicated, with one copy assigned to the full deliberative schema and one to the representative deliberative schema. This means that for every agent in Full there is a duplicate of that agent in Rep that has the same set of starting reasons. Creating agents this way better enables a direct pairwise comparison of the two groups, since the initial epistemic makeup of the agents in Full and Rep is exactly the same.

In each round of full deliberation, an agent is chosen at random and asked to share a reason randomly selected from among the *m* reasons they possess. When a reason is shared in Full every other agent incorporates it into its headspace so long as it isn’t already there. This deliberative process repeats for *2t* rounds. In Rep, by contrast, agents are first broken into *g* smaller “cells” before reason sharing begins. Evidence shared in a cell is only heard by other members of that cell. Then, for each round of Rep, like in Full, agents in the cells share a random piece of evidence, but in the representative case, each of the cells deliberates in parallel. After *t* rounds, one representative agent is generated by each of the *g* cells. The representative agents are new agents created by collecting evidence from among the weightiest evidence of each cell—like the neutral deliberators of the planning cells.

**Fig 1:**
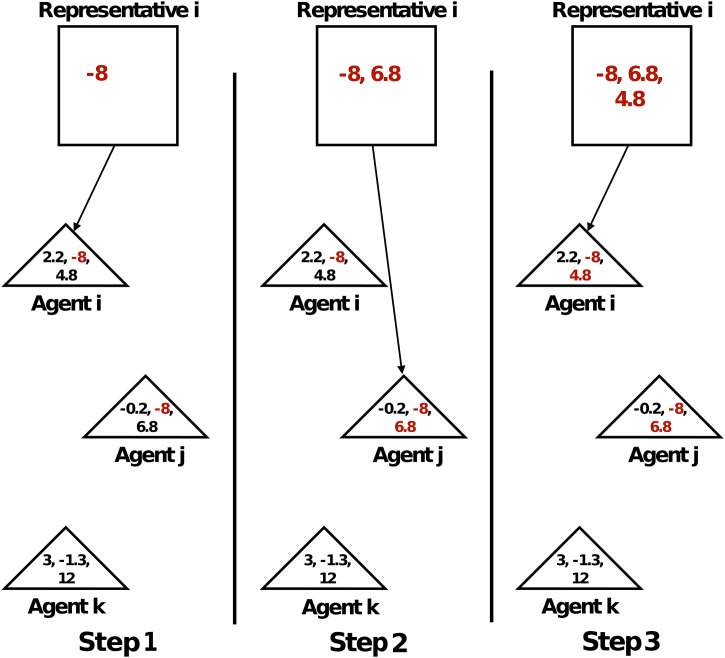
An illustration of how representatives are generated using a case of three agents per cell, each with three reasons. The representative chooses an agent at random and selects its strongest/weightiest reason that the representative agent has not already “heard”. This goes on until the representative agent has as many reasons as each agent in the cell started with.

Each representative agent fills its headspace by randomly prompting a constituent for its strongest reason that it has not yet heard. This process repeats until the representative agent has *m* reasons—the same number of reasons that each agent started with—yielding *g* representatives, each with *m* reasons (Fig 1). These representative agents then continue deliberating among themselves for an additional *t* rounds. In this way representative agents in our model are naturally representative of the deliberative body, since all their reasons come from their constituents, but like real representatives, they don’t possess all the evidence of their constituents [15]. This process tracks the broader method of information sharing in the model as well as our epistemic interests, by seeding representatives with the best reasons available. Note that by forming representatives this way, it is possible, though improbable, for there to be reasons had by the group that are stronger than those held by the representative. In practice, however, the results are robust to this modeling choice.

**Fig 2:**
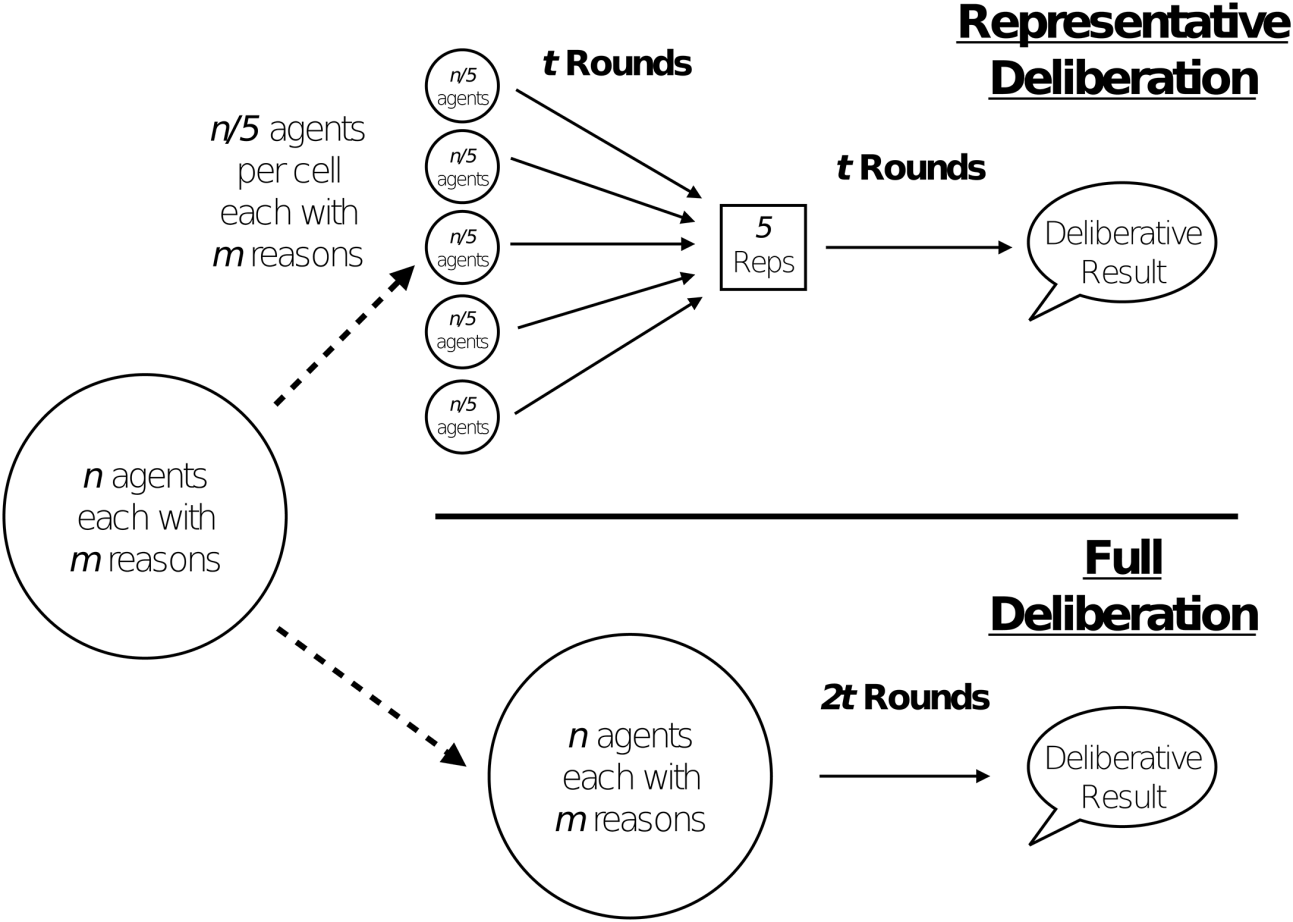
Illustration of model procedure with five cells (g = 5).

In contrast to the full schema where all agents exchange reasons together for *2t* rounds, agents in the representative mode deliberate in their cells for *t* rounds and then the *g* representative agents continue to deliberate for another *t* rounds. This ensures that agents in both Full and Rep hear the same number of reasons in the same number of deliberative rounds. For a given run we compare *g* representative agents to the success of all *n* agents in the full deliberation mode. Importantly then, this means that the only differences between Full and Rep are (1) how the agents are initially grouped and (2) whether, after some number of rounds (*t*), a small number of representative agents take up deliberation in lieu of their constituents (Fig 2).

Notice that by structuring the model this way, there’s a sense in which we have stacked the deck in favor of full deliberation. In Full, all agents are connected to one another and don’t have epistemic limitations, so their beliefs are a perfect reflection of all the evidence they’ve received. In contrast, Rep only passes on a portion of its constituents’ reasons to the representatives. Prima facie, this leaves Full with more available evidence, which seems like it should result in greater epistemic success. Moreover, representative agents aren’t programmed to be ‘smarter’ than their constituents. The difference is purely structural, not epistemic.

We also run the model under different memory conditions. At first we run the model without any limitations on the number of reasons agents are able to hold—we call this the unlimited memory condition. As agents share evidence each agent in the group retains every reason they hear. In contrast we also run the model where agents have limited memory. Here agents are only able to retain a fixed number of reasons after which they must discard evidence to make room for newer reasons they encounter. We instantiate limited memory two ways, what we call “weight-minded forgetting” and “coherence-minded forgetting” [53]. In weight-minded forgetting agents keep their weightiest reasons, discarding the lowest weighted reason available to them (including the one they have just heard). For example, if the agent has a memory limited to 7 reasons, when the agent gets an 8th reason they’ll lose the reason among the 8 with the smallest magnitude (the one with the lowest absolute value). In the second way, coherence-minded forgetting, the agents prioritize reasons for the view that is best supported by all their reasons. When such an agent gets an 8th reason that goes over their memory limit, they prefer to forget a reason that runs contrary to the view that is supported by all 8 of their reasons. So if the agent’s belief was positive, they would forget the negative reason with the smallest magnitude. (If all their reasons were positive they would just discard their weakest reason.) This method tracks how well agents balance epistemic considerations given their current weighted beliefs.

Each model run contains a different set of 500 reasons for 169 agents making for 13 cells, repeated 1,000 times for each parameter setting. Each agent is initially given 7 reasons. We measure success by comparing the percentage of the agents in Full and Rep that are on the side of the truth at time *2t* (when both groups have stopped deliberating)*.* We compare how the two modes fare as a function of four conditions: (1) length of deliberation (number of model steps), (2) evidentiary heterogeneity (variance of the evidence), (3) problem difficulty (success as the balance of the evidence tends towards zero, |Σ*r* |→ 0), and (4) memory constraints (unlimited evidence retention versus weight and coherence minded forgetting).

## 3. Results

Broadly speaking, we find that representation shines in shorter deliberative sessions, when there is more widely distributed evidence, with difficult problems, or when agents can’t hold on to all the evidence they encounter. Section 3.1 presents three key findings. First, Rep outperforms Full under time pressure. Second, Rep performs comparatively well when evidence is highly dispersed. Third, Rep possesses an advantage on difficult problems, even in long deliberative sessions. Each is the case when agents have unlimited memory. In Section 3.2 we show that when agents have memory limitations, specifically coherence-minded limitations, Rep almost always does better than Full.

These results show the epistemic strength of representation in two ways. First, as constraints come to track those of the real-world and agents benefit from triaging weightier evidence, representative structures perform better. And second, the epistemic benefits of representation result from the structure of the deliberative process rather than from any epistemic gifts endowed to representative agents by the model. While reading, we invite the reader to consult Table 5, provided in Section 4, which provides a summary of the results discussed.

### 3.1. Success with unlimited memory

We start by considering the simplest cases, where agents remember everything they hear and there is an evidentiary variance of 1. Focusing on the length of deliberation, the key pattern is straightforward: Rep performs better in shorter deliberative sessions. In deliberations of 5 time-steps, Rep does about 4% better than Full on average. At 30 steps, Rep still performs better, but only by 0.7%. At 100 steps, however, Full has the advantage and does about 4.6% better, and at 1,000 steps Full does about 6.3% better. In cases with maximal constraints on the period of deliberation, Rep consistently outperforms Full. As time pressures diminish and the deliberative period lengthens the result reverses and Full beats Rep.

The second condition we test is evidentiary heterogeneity—situations in which a small number of observations carry disproportionate weight. What we find is that an increase in evidentiary heterogeneity systematically advantages representative deliberation in contrast to full participation. As the variance of evidence increases, Rep outperforms Full for longer periods of deliberation, and in the highest-variance condition we examine ($\alpha /\beta^{\text{2}}$ = 100) Full never surpasses Rep within 1000 steps. Table 1 illustrates this pattern by reporting the “cross-over” point at which Full begins to outperform Rep at different levels of variance. Because outcomes are measured at discrete intervals (5, 10, 20, 30, 50, 100, 200, 1000 steps), these cross-over values represent the earliest observed point of reversal rather than exact thresholds. As variance declines—that is, as evidence becomes more narrowly distributed—Full overtakes Rep more quickly, and its advantage becomes more statistically stable across runs. Taken together, these results indicate that representative structures are particularly effective in high-variance informational environments, where a few pieces of evidence dominate the evidentiary landscape.

**Table 1:** Relative deliberative superiority of full deliberation under unlimited memory. The second column reflects the point at which Full outperforms Rep for a given variance. In the third column, the first number is the percentage of runs in which Full is better than Rep at 1,000 steps of the model and the second number in parentheses is the t-statistic indicating how stable the result is.

Variance	Cross-over when Full Beats Rep	Amount Full Better at 1000 Steps
100	N/A	0% (N/A)
10	200 steps	0.05% (0.2)
1	50 steps	6.33% (9.4)

Because the representative agents in Rep aggregate information by selecting from the strongest reasons of their constituents, the deliberative structure provides search efficiencies that make representatives both faster and better able to evaluate widely dispersed evidence. But Rep tends to underperform in the long run here because it doesn’t have access to all of the reasons that Full does. Both time and evidentiary heterogeneity play a role, but as the length of the deliberative period increases, the relative importance of variance swamps that of time. An ANOVA of the relationship between the variance of the evidence and deliberative length on the one hand, and epistemic success on the other, indicates that both of the former parameters are highly significant (F = 2027 and 18, respectively), though the relative importance of variance is much greater than deliberative length.

We next consider a third variable: problem difficulty, namely how deliberation performs as the balance of the evidence tends towards 0. To do this, it will be important to distinguish between the weight of the evidence and the difficulty of the problem. Imagine two equally difficult problems balanced by evidence with very different evidentiary spreads, e.g., {100, −100.1} and {1, −1.1}. While the evidence in the first set is weightier, both cases are more difficult than an evidentiary set of {1, −2}, since there is less margin for error in the former. Although heterogeneity and difficulty are related (they are correlated, as less heterogeneous evidence tends to have greater difficulty), they are conceptually and statistically distinct. When we regress representative epistemic success on both evidentiary heterogeneity and difficulty we find that difficulty is significantly associated with Rep success, even while controlling for heterogeneity (Table 2). The key takeaway is that Rep is better than Full when it comes to difficult problems.

**Table 2:** Two model fits of Rep’s advantage in subtle cases. The positive value on *Difficulty* indicates that Rep performs marginally better as the absolute value of the truth goes to zero, while *Variance* is a measure of the variance parameter. The response variable is the success of Rep minus the success of Full.

	OLS	Ordered Logit
	(1)	(2)
Variance	0.018^***^	0.121^***^
	(0.001)	(0.007)
Difficulty	0.0002^***^	0.001^**^
	(0.0001)	(0.0004)
Constant	0.015^***^	
	(0.003)	
Observations	56,000	56,000
R^2^	0.004	
Adjusted R^2^	0.004	
Residual Std. Error	0.413 (df = 55997)	
F Statistic	121.148^***^ (df = 2; 55997)	

Note: *p < 0.1; **p < 0.05; ***p < 0.01.

This is a surprising result. Prima facie we might think that Full would excel in cases where the truth turned on subtleties resolved by small pieces of evidence. Since Full never loses evidence it should outperform Rep. But this isn’t the case. The mechanism driving this result is Full’s overfitting. For each run, every agent is randomly given *m* reasons from among *r* and then the whole group is duplicated, so that the agents running in the full configuration have the same epistemic profiles as those running in the representative configuration. What happens, especially in cases of low evidentiary variance, is that Full overfits agents’ beliefs to the available evidence, even though the truth is determined by the complete set of reasons.

By triaging reasons in favor of the stronger ones, Rep more efficiently filters the evidence so that the deliberative outcome isn’t overfit. It’s the deliberative structure of the representative group that gives rise to its advantage, not any epistemic advantage of individual representative agents. This is one of the central findings of the paper: representative deliberation performs better precisely in cases where the truth turns on subtle differences in evidence.

### 3.2. Success with limited memory

So far, we have compared Full and Rep under the assumption that the agents have unlimited memory. With no memory constraints Rep performs better under time pressure and as information is more widely dispersed; Full does better with more time and less spread of the evidence. But in reality, people can’t remember every piece of evidence they encounter. Even gifted people forget a considerable amount. In order to track these more realistic deliberative dynamics, here we run the model by regulating agents’ memories using weight-minded and coherence-minded methods. We model cognitive capacity as agents having a memory limit of seven reasons, and they discard one reason when they encounter an eighth. Although the results are robust to a range of memory capacities, we chose to use seven memory slots in line with Miller [59] (see also Shiffrin and Nosofsky [60]). The key finding here is that once agents have memory constraints, Rep almost always matches or outperforms Full.

For weight-minded agents, Full almost never outperforms Rep. With fewer steps to deliberate, Rep does considerably better (Table 3), but in longer deliberative sessions Rep and Full tie at every tested variance. Rep still has a marginal advantage at higher variances, obtaining more accuracy in fewer time-steps, but with a long enough period of deliberation, the two groups perform the same. Results for coherence-minded forgetting are more stark. Here Rep outperformed Full in all but two of 56,000 runs (Table 4). In the two outlying runs where Full performed better, the difference was still not significant.

**Table 3:** The superiority of representative deliberation at 5 deliberative steps for weight-minded memory management. We show results for short deliberative periods to accentuate the success of Rep given varying degrees of evidentiary variance. Ultimately, for long deliberative periods given weight-minded forgetting, Full and Rep come to tie on epistemic success. In the second column, the first number is the percentage of runs in which Rep performs better and the second number in parentheses is the t-statistic indicating how stable the result.

Variance	Amount Rep Better at 5 Steps
100	13.25% (13.2)
10	9.59% (10.4)
1	2.75% (3.0)
0.2	1.36% (1.4)

**Table 4:** Superiority of representative deliberation at 1000 steps using coherence-minded limited memory management. In the second column, the first number is the percentage better and the second number in parentheses is the t-statistic indicating how stable the result is at 1,000 steps of the model.

Variance	Amount Rep Better at 1000 Steps
100	0.74% (1.3)
10	3.59% (3.4)
1	3.11% (3.1)
0.2	4.82% (5.7)

The reason for Rep’s strength with weight-minded memory management is straightforward. Representative agents are created by sampling from among the strongest evidence of their constituents, which is a close approximation of the weight-minded memory management mechanism. Without memory limitations, Full can do better because Rep loses information when the representative agents are created, overlooking less weighty evidence. But here, under weight-minded forgetting, all the agents are using the same method by which representative agents are generated, leading Full and Rep to tie.

The story is more interesting for coherence-minded agents, however. While Rep does worse under coherence-minded forgetting than under weight-minded constraints, Full does worse still. Rep’s advantage here doesn’t come from the fact that representative agents select for weighty reasons, either. To test this we ran experiments where instead of creating representative agents in the normal way, reasons were selected according to the “coherence-minded” rule. Here, rather than selecting agents at random and asking for their strongest reason which the representative had not yet heard, the representative selects an agent at random and asks them for their strongest reason and then asks all subsequent randomly chosen agents for their strongest reason conforming to the extant belief of the representative. (If the subsequent randomly chosen agent didn’t have a reason supporting the representative’s belief they were told to share their weakest reason opposed.) Comparing weight-minded to coherence-minded representative creation we found no significant difference between the outcomes, indicating that the difference between Full and Rep results from the second round of deliberation. Representatives’ reason-gathering process isn’t what gives them an edge here.

The mechanism here for Rep’s advantage differs from the cases of unlimited memory and turns on how representation restructures deliberation. Both full deliberation and cell deliberation—the small deliberative groups that pass up reasons to representative agents for the second stage of Rep deliberation—perform roughly as well as one another, coming to equilibrium quickly, generally in fewer than 10 time-steps. And once equilibrium is reached, no agent changes their mind—deliberating agents in both the full configuration and in the cells never see a belief change after 90 time-steps. However, when representatives take over for agents in cells, the deliberation gets “unstuck” and evidence sharing resumes, improving their truth-tracking by about 2.3% over the initial ten time-steps. Since coherence-minded agents are apt to polarize [53]—both those in cells and full deliberation alike—the reconfiguration of reasons during the generation of representative agents helps reset deliberation in entrenched cells, allowing Rep to consistently outperform Full. The key takeaway is that representation introduces a second phase of deliberation that can jumpstart stalled or polarized groups, allowing continued information exchange where Full has already converged.

## 4. Conclusion

Deliberative events, like the 1987 German ISDN Planning Cell forum, offer instances where ordinary citizens discuss pressing policy matters and offer recommendations to policymakers. Beyond these political cases, many corporations and firms rely on hierarchy to aggregate insights upward [61,62]. Rather than imposing a more restricted and hierarchical deliberative process, where a select group of deliberators feed recommendations up to a further level of decision making, a plenary body could deliberate on all matters at once. Under those circumstances, full deliberation would have access to all the evidence the representative deliberators do and very possibly more. Thus, as we mentioned in the introduction, it is natural to think representative deliberation would be at a disadvantage [7,63].

The ABM here serves as a minimal model of deliberative bodies, allowing us to “open the hood” of hierarchical, representative information aggregation. Table 5 serves as a good overview of our results. Surprisingly, our results show that representation can endogenously produce better epistemic outcomes in virtue of the structural features of the deliberative institution, rather than from the selection of epistemically superior representative agents. Here we find that in cases where the length of deliberation is limited, when there are a few strong reasons, when the problems are difficult, and when deliberation comes to an early deadlock, there are good reasons to embrace a representative structure for deliberation. While Full fares best in the long run without memory limitations, Rep demonstrates advantages in shorter deliberative sessions and when a few distinct pieces of evidence have outsized importance on the truth. And when agents face memory limitations, Rep consistently performs as well as or better than Full. Yet representation also fares better with difficult problems, even under ideal conditions, in the sense that Rep does consistently better at arriving at the truth when the balance of evidence is near zero, independent of time or memory constraints (Table 5).

**Table 5:** Summary of results from Section 3.

	No memory constraints	Memory constraints (coherence)
**Length of deliberation**	Full is never worse by 1000 steps	Rep is better at all lengths of deliberation, and as much as ~5% better at shorter lengths
**Evidentiary heterogeneity**	As heterogeneity increases it takes longer for Full to outperform Rep	Rep is comparatively better the greater the heterogeneity
**Problem difficulty**	Rep is significantly better than Full for greater difficulty	No difference
**Color legend:**		
	Full better	
	Mixed	
	Rep better	

We began with a research question to understand how representation might outperform full, plenary deliberation. We find two mechanisms that explain cases of representation’s superiority in our model. The first regards representation’s need to triage evidence. Because representation doesn’t have the luxury of making decisions with all the available evidence, it must cull the evidence at some point in the deliberative process. It does so by having the representative agents select the weightier evidence from among their constituents, leaving behind less weighty reasons. Either because the weightiest evidence serves as a lodestar for the truth or because smaller bits of evidence can lead to overfitting, representation’s knack for grabbing the weightiest evidence can allow for more reliable convergence on the truth. Representation shines in model runs where weightier evidence is a good heuristic for the truth, but also in difficult problems where overfitting to the available evidence is actually suboptimal. Notably, difficult problems here need not be thought of as unimportant ones. They can be cases where the margin of the evidence for and against is narrow, but the stakes of the deliberation are still high. While full deliberation has access to more evidence, it is still only a subset of the full set of reasons. Because of that, full deliberation overinterprets its available evidence while representatives resist overfitting. Surprisingly, restricting the set of evidence is a better way of truth tracking as problems become more difficult.

The second mechanism by which representation succeeds is by introducing a second phase into the deliberative process. In coherence-minded memory management cases, representation can prevent groups from getting stuck on inferior solutions when a better alternative exists—adding to a prominent modeling literature which similarly finds that searches that take time to converge on an optimum outperform those that do so quickly [52,64–66]. Yes, full deliberation has the benefit of brute force search, combing through all the available evidence. But when agents are only able to hold a limited set of evidence, the group can arrive at an imperfect consensus relatively quickly. In these cases, representation has the ability to reorder the available evidence and free polarized deliberation by resetting deliberation at another tier, allowing convergence on better outcomes. Merely changing the structure of deliberation helps groups move past some forms of polarization. Each of these conditions—length of deliberation, evidentiary heterogeneity, problem difficulty, and memory constraints —illustrate how representation can shine in virtue of its deliberative structure.

Not every representative system is built similarly, so our results won’t shed light on all of them. Nevertheless, our ABM here offers a simple model of deliberation that can help us understand some ways that representative deliberation can outperform full deliberation. Of course, in the real-world there are many practical reasons why representation is preferable. Here, though, we demonstrate why even in theory we can expect representation to be superior sometimes on structural merits alone.
